# Frequency and determinants of health care utilization for symptomatic reproductive tract infections in rural Indian women: A cross-sectional study

**DOI:** 10.1371/journal.pone.0225687

**Published:** 2019-12-05

**Authors:** Mitchell A. Kinkor, Bijaya K. Padhi, Pinaki Panigrahi, Kelly K. Baker

**Affiliations:** 1 Carver College of Medicine, University of Iowa, Iowa City, Iowa, United States of America; 2 Asian Institute of Public Health, Bhubaneswar, Odisha, India; 3 Post Graduate Institute of Medical Education and Research (PGIMER), Chandigarh, India; 4 University of Nebraska Medical Center, Omaha, Nebraska, United States of America; 5 College of Public Health, University of Iowa, Iowa City, Iowa, United States of America; ESIC Medical College & PGIMSR, INDIA

## Abstract

**Introduction:**

The public health burden of reproductive tract infections (RTIs) among women in rural areas of low-income countries is poorly addressed because health care seeking for treatment of RTIs is inadequate. There are gaps in knowledge about whether low care seeking behavior stems from challenges in accessing health care versus women's recognition of and response to RTI-specific disease symptoms. We aim to identify determinants of care seeking behavior and analyze the difference in utilization of health care resources in response to symptoms of an RTI versus non-RTI disease symptoms in rural India. This will aid in the design of interventions that promote RTI care seeking behavior.

**Methods:**

Our analysis uses data from a cross-sectional, population-based surveillance survey among rural, non-pregnant women in Odisha, India, from 2013–2014 (n = 3,600). We utilized bivariate logistic regression to determine the degree that certain determinants are associated with a woman’s likelihood to seek RTI treatment, and chi-Squared tests to assess for differences in health care resources used for non-RTI versus RTI symptoms.

**Results:**

Married women were significantly more likely to seek health care for RTI symptoms (Odds Ratio (OR) = 1.9, 95% Confidence Interval (CI): 1.2–3.0) while unmarried adolescents were less likely to seek treatment (OR = 0.4, CI: 0.2–0.6). There was no association between RTI health care seeking with education level, belief about whether symptoms can be treated, or poverty. The majority (73.8%) of women who did not seek treatment for RTI symptoms reported not seeking treatment because they did not know treatment was needed. Women utilized formal health care providers at a higher rate in response to RTI symptoms than in response to their most recent symptoms of any kind (p = 0.003).

**Conclusions:**

Community-based reproductive health education interventions are needed to increase health care seeking behavior for RTIs in rural Indian women. Interventions should target unmarried women and focus on both sexual health education and access to care.

## Introduction

Millions of women worldwide are impacted by reproductive tract infections (RTIs), a group of infectious and non-infectious diseases that all impact reproductive tract physiology and immunology and, as a result, share common symptoms, such as vaginal itching and discharge. RTIs carry an increased risk of pelvic inflammatory disease, spontaneous abortion, preterm delivery, and human immunodeficiency virus infection [1–5]. Bacterial vaginosis (BV) is the most common RTI in India, with a prevalence estimated between 16%-19% in one rural Indian community-based survey [6]. Urinary tract infections (UTIs) and other RTIs, like candida infections, are common in Indian women as well, though sexually transmitted infections (STIs) are less prevalent RTIs in Indian women with a rate estimated from 3%-5% [6]. The overall high prevalence of RTIs highlight that many women in India are at risk for severe, long-term diseases. Much of that secondary disease risk can be avoided by seeking treatment from a healthcare provider. While there is no guaranteed cure for many RTIs, the infections can be effectively treated with either probiotic or antimicrobial medications [7–9]. Treatment can be especially effective in preventing long term side effects if it is detected in its early stages, which further stresses the importance of improving treatment seeking behavior amongst both symptomatic and asymptomatic patients [9, 10].

Assessment of District Level Household Surveys (DHS) suggests that only 40% of Indian women seek medical care when RTI symptoms are recognized [11]. Recent literature has suggested that rate may be as low as 33% in rural areas [11–14]. Though RTIs can cause symptoms, it is challenging for women to detect RTIs on their own because over half of RTI cases can be asymptomatic [12, 15, 16]. When symptoms are recognized, factors such as cultural shame associated with RTIs, personal reproductive health education, life stage status, autonomy in personal health decision making, wealth, and ability to access healthcare resources are associated with a woman’s likelihood to seek medical treatment for gynecological issues [12]. Interventions aimed at increasing treatment seeking behavior for RTI symptoms should be targeted at key barriers to gynecological health care utilization. However, a great deal about women’s reproductive health care seeking behavior, especially in rural areas where formal providers are limited, remains unknown. Many studies of RTIs have centered on women’s use of formal medical providers. This may be underestimating health care utilization because many women, especially rural, may purposely seek care for RTIs via alternative providers, such as traditional healers, or home remedies, because these options may be more private, accessible, or affordable [10]. Interventions may need to include informal providers in health education and service delivery to improve the timely utilization of formal gynecological services among some groups of women. Additionally, many previous studies that have focused on care seeking behavior for RTI symptoms have not considered the general health care seeking behavior of their respondents. Observing the difference between patients’ treatment seeking behavior for RTI symptoms versus other non-RTI disease symptoms could provide insight into the relationship that Indian women have with their public health system for gender-specific diseases, along with guidance regarding the design of effective interventions.

The first objective of this study was to identify the prevalence of health care seeking behavior for rural Indian women in the Odisha area with recent RTI symptoms and identify determinants of care seeking behavior among women with recent symptoms. Second, we aimed to describe the type of providers Indian women seek to treat those symptoms. Finally, we aimed to analyze the difference of utilization of health care resources in response to RTI symptoms and non-RTI symptoms.

## Materials and methods

### Study design and sample size

A cross-sectional, population-based surveillance survey investigating demographics, WASH (Water Sanitation and Hygiene) habits, gynecological health, and health care seeking behavior in response to symptoms was conducted between September 2013 and March 2014 in Odisha, India [17]. For our analysis, 3,600 non-pregnant women who reported experiencing menstrual periods and were between the mean age of menarche (13.6 years) and menopause (46.1 years) for Indian women were considered eligible. Thirty women (0.83%) were excluded from analysis due to incomplete responses, and 3 were excluded for data quality issues, leaving 3,567 responses to analyze. All 3,567 women were asked if they experienced two common RTI symptoms (vaginal itching and discharge) within the last two weeks to establish 2-week prevalence of RTI symptoms. If they responded yes, they were asked if they sought treatment for those symptoms and where they sought care. All respondents were asked the same questions about care-seeking relating to the last time they experienced symptoms of any kind. All women who experienced RTI symptoms in the two weeks prior to the survey were considered eligible for a study on RTI health care seeking behaviors. Women who did not self-report recent RTI symptoms were not included in bivariate analysis of determinants for RTI health care seeking behavior, though their responses were used in analysis of the utilization of formal and informal health care providers. More information on study setting, ethical considerations, data collection, and data management of the survey has been previously reported [17].

### Human subjects ethical approval

Written informed consent was obtained from all respondents prior to data collection. The study was approved by the scientific and ethical review committees at the Asian Institute of Public Health (ERC Protocol No. 2013–03) and Emory University (Protocol 00069418).

### Outcome

Self-reported health care seeking behavior for treatment of acute self-reported RTI symptoms is the outcome of interest for understanding the determinants of health care utilization

### Variable selection

The potential determinants of both RTI symptoms and health care seeking behavior in response to those symptoms were considered through literature review [6, 10, 12–14, 18, 19] during our variable selection process. Our literature review indicated that key determinants of women’s reproductive health utilization in India included life stage status, poverty, education level, and belief that RTI symptoms can be prevented. Proxy variables designed to account for life stage status [17], wealth [20], and education are described in Table 1.

**Table 1:** Definition of variables.

Category	Variable	Definition
**Life stage status**	Unmarried youth	Single and less than 24 years of age
	Newly married	Married for 2 or less years
	Established married	Married for more than 2 years
	Other	Single/divorced/widowed/separated and/or over 24 years of age
**Poverty**	BPL card	Possession of a Below Poverty Line (BPL) card
	Lives in home with more than 2.5 people/sleeping room	Lives in home with an above median number (2.5) of people/sleeping room
**Education**	No formal education	
	Primary education	Completed some primary education
	Secondary education	Completed some secondary education

### Statistical methods

Data were analyzed using Stata 15.1 (StataCorp, College Station, TX). First, we compared the frequency of demographic variables among women who reported RTI symptoms to women who did not using Chi-squared analysis to understand whether the RTI symptomatic population was representative of the population at large. Among women who reported experiencing RTI symptoms, bivariate logistic regression was used to compare the odds of treatment seeking behavior in response to RTI symptoms for categories of each determinant variable. A fully adjusted logistic regression model was constructed for any variables associated with RTI health care seeking behavior at p<0.2. Descriptive statistics were quantified for the prevalence of care seeking behavior and the self-reported reasoning why care was not sought, if it was not sought. We tested for differences in types of formal provider utilization for non-RTI symptomatic survey respondents’ most recent symptoms of any kind and RTI symptomatic survey respondents’ RTI symptoms using Chi-Squared analysis. It is possible that the survey respondents without RTI symptoms in the last two weeks could have reported on health care use for RTI symptoms (vaginal itching or discharge) occurring more than two weeks prior to the survey date, although this is likely a small proportion of our sample.

## Results

### Demographics of respondents with 2-week prevalence of RTI symptoms

Of the 3,567 non-pregnant respondents, 342 (9.6%) women self-reported symptoms of a RTI (abnormal vaginal itching and discharge) in a 2-week window before the survey date (Table 2). Some demographic groups were more likely to report the presence of RTI symptoms in our survey [17], so the study population of women with recent symptoms reflect increased sampling from those groups with higher rates of RTI symptom reporting relative to the overall population. Specifically, respondents who reported RTI symptoms were more likely to be Muslim or housewives, and to have a higher mean number of minutes of travel required to reach a primary health care center. Those who self-reported RTI symptoms showed a greater tendency to have sought health care for their most recent symptoms of any type (non-RTI or RTI) than women without recent RTI symptoms. The respondent’s state of origin, education level, life stage status, and caste were not significantly different between those who reported RTI symptoms and those who did not, though the plurality of respondents refused to provide their caste.

**Table 2:** Demographic differences between women reporting acute RTI symptoms within the last two weeks versus women with no symptoms in the last two weeks.

	Level, sample size	% of women who reported RTI symptoms (N = 342)	% of those who did not report RTI symptoms (N = 3, 225)	P
**District**				**0.092**
	Khordha n = 998	31.9% (n = 109)	27.6% (n = 889)	
	Sundergarh n = 2,569	68.1% (n = 233)	72.4% (n = 2,336)	
**Religion**				**0.000**
	Hindu, n = 2,505	72.2% (n = 247)	70.0% (n = 2,558)	
	Christian, n = 863	15.8% (n = 54)	25.1% (n = 809)	
	Muslim, n = 188	11.7% (n = 40)	4.6% (n = 148)	
	Other religion, n = 11	0.3% (n = 1)	0.3% (n = 10)	
**Occupation**				**0.005**
	Employed, n = 429	8.8% (n = 30)	12.4% (n = 339)	
	Housewife, n = 2,020	64.6% (n = 221)	55.8% (n = 1,799)	
	Student, n = 589	11.7% (n = 40)	17.0% (n = 549)	
	Other occupation, n = 529	14.9% (n = 51)	14.8% (n = 478)	
**Caste**				**0.087**
	Other backwards caste, n = 627	19.6% (n = 67)	17.4% (n = 560)	
	Other caste, n = 270	10.5% (n = 36)	7.3% (n = 234)	
	Scheduled caste, n = 652	14.9% (n = 51)	18.6% (n = 601)	
	Scheduled tribe, n = 956	27.2(n = % (n = 93)	27.8% (n = 863)	
	Caste not reported, n = 1,062	27.8% (n = 95)	30.0% (n = 967)	
**Education**				**0.843**
	No formal education, n = 661	19.0% (n = 65)	18.5% (n = 596)	
	Primary education, n = 645	19.0% (n = 65)	18.0% (n = 580)	
	Secondary education, n = 2,261	62.0% (n = 212)	63.5% (n = 2,049)	
**Life Stage Status**				**0.091**
	Unmarried youth, n = 1,166	27.5% (n = 94)	33.2% (n = 1072)	
	Newly married, n = 74	2.9% (n = 10)	2.0% (n = 64)	
	Established married, n = 2,132	64.9% (n = 222)	59.2% (n = 1,910)	
	Other, n = 192	4.7% (n = 16)	5.5% (n = 176)	
**Wealth**				** **
	BPL Card, n = 1,878	52.1% (n = 178)	52.7% (n = 1,700)	**0.814**
	Lives in home with more than 2.5 people/room, n = 1,491	42.4% (n = 145)	41.7% (n = 1,346)	**0.814**
**Sought care during last sickness (any symptoms)**		59.6% (n = 204)	34.7% (n = 1,120)	**0.000**
**Belief that RTI symptoms cannot be prevented**		1.8% (n = 6)	2.1% (n = 67)	**0.688**
**Mean age—years (SD)**		27.3 (8.2)	26.5 (8.0)	**0.082**
**Mean distance from primary health care center (SD)**		51.5 minutes (30.3)	45.7 minutes (25.3)	**0.000**

### Determinants of gynecological health care seeking behavior in response to RTI symptoms

Of the 342 respondents that self-reported RTI symptoms in the last 2 weeks, 161 women (47.1%) reported that they sought care for those RTI symptoms (Table 3). Status as an established married woman versus all other life stages (OR = 1.9, CI: 1.2–3.0) was a significant predictor of health care seeking behavior for RTI symptoms in bivariate analysis. Status as an unmarried adolescent was significantly associated with less health care seeking behavior for RTI symptoms (OR = 0.4, CI: 0.2–0.6). Indicators of poverty (BPL Card, more than 2.5 people per sleeping room), a woman’s education level, and belief that symptoms could not be prevented were not statistically associated with RTI care seeking. A multivariable model was not constructed since life stage was the only variable that met inclusion criteria.

**Table 3:** Factors associated with health care utilization for RTI symptoms among symptomatic women.

Determinant level, sample size			Proportion of care seekers, N = 161	Proportion of non-care seekers, N = 181	Bivariate model OR (95% CI)
**Education**					
	No formal education, n = 65	20.5% (n = 33)		19.9% (n = 32)	1.2 (0.7, 2.1)
	Primary education, n = 65	19.9% (n = 32)		20.5% (n = 33)	1.1 (0.6, 1.9)
	Secondary education, n = 212	59.6% (n = 96)		72.0% (n = 116)	0.8 (0.5, 1.3)
**Health care**					
	Believe RTI symptoms cannot be prevented, n = 6	0.6% (n = 1)		2.8% (n = 5)	0.2 (0.0, 1.9)
**Wealth**					
	BPL card, n = 178	49.7% (n = 80)		54.1% (n = 98)	0.8 (0.5, 1.3)
	Lives in home with more than 2.5 people/room, n = 145	41.6% (n = 67)		43.1% (n = 78)	0.9 (0.6, 1.4)
**Life Stage Status**					
	Unmarried youth, n = 94	18.0% (n = 29)		35.9% (n = 65)	0.4 (0.2, 0.6)
	Newly married, n = 10	3.7% (n = 6)		2.2% (n = 4)	1.7 (0.5, 6.2)
	Established married, n = 222	72.3% (n = 117)		58.0% (n = 105)	1.9 (1.2, 3.0)
	Other, n = 16	6.0% (n = 9)		3.9% (n = 7)	1.5 (0.5, 4.0)

### Cited reasons for non-health care seeking

Among those women who did not seek treatment for RTI symptoms, the overwhelming majority (73.8%) reported that they did not think they needed treatment for the described symptoms, followed by inability to take time off from work (8.5%), and health center being too far from their home (5.7%) (Table 4). After knowledge of the severity of reproductive symptoms, the next four most common reasons for declining to seek treatment for RTI symptoms were all directly related to health care accessibility in the area.

**Table 4:** Reasons Why RTI symptomatic women (N = 141) Did Not Seek Treatment for RTI Symptoms.

Reason	% (n)
I did not think I needed treatment	73.8 (104)
Could not take time away from work	8.5 (12)
Clinic too far from home	5.7 (8)
Unable to find transport	2.8 (4)
Cost for travel too high	3.5 (5)
Children could not be left home alone	2.8 (4)
Did not have permission from husband/mother-in-law/other	2.1 (3)
Cost for treatment too high	0.7 (1)
Flood or bad weather	0.0 (0)
Not happy with clinical services in area	0.0 (0)

### Health care providers for RTI and non-RTI symptoms

Women who sought care for RTI symptoms were more likely to utilize formal providers (hospitals, community health centers) compared to women without RTI symptoms but who reported seeking treatment the last time they were sick with any symptoms (Table 5,77.5% versus 67.2%, p = 0.003). The individual types of formal and informal providers (traditional healers, market remedies, friends/relative, religious leader, pharmacy) were used at similar frequencies. For both RTI symptoms and non-RTI symptoms, major hospitals were the most common health care provider utilized, though they were more commonly utilized in specific response to RTI symptoms and community health centers were more commonly utilized in response to any symptom. Informal providers [21] were more frequently utilized by women reporting treatment seeking for non-RTI symptoms of any kind mainly due to their high utilization of the pharmacy for treatment. Traditional healers, religious leaders, and friends/relatives were visited for treatment at similar rates between the groups.

**Table 5:** Care provider sought for RTI symptoms among RTI symptomatic women vs. RTI asymptomatic women seeking care for other recent non-RTI symptoms.

RTI symptomatic women, N = 129 ^a^	% (n)	RTI asymptomatic women with other recent symptoms, N = 1,117 ^b^	% (n)	P-Value (formal vs informal)
**Formal Provider** - Major hospital - Community health center	**77.5 (100)** 57.4 (74) 20.2 (26)	**Formal Provider** Major hospital - Community health center	**67.2 (751)** 33.8 (378) 33.4 (373)	
**Informal Provider** - Pharmacy - Traditional Healer - Remedy purchased at market - Friend/Relative - Religious leader	**22.5 (29)** 10.1 (13) 5.4 (7) 3.1 (4) 2.3 (3) 1.2 (2)	**Informal Provider** - Pharmacy - Traditional Healer - Remedy purchased at market - Friend/Relative - Religious leader	**32.8 (366)** 21.9 (245) 6.4 (71) 2.0 (22) 1.7 (19) 0.8 (9)	
**0.003**				

^**a**^ 32 respondents indicated that they sought care but did not report location.

^b^ 3 respondents indicated that they sought care but did not report location.

## Discussion

This study sought to identify the prevalence of health care utilization for RTI symptoms in rural Indian women, the determinants of gynecological health care seeking behavior, and the relationship between RTI symptom-specific health care seeking behavior and health care seeking behavior in response to any other type of disease symptom. First, we showed that half of all symptomatic women did not seek health care for RTI symptoms, primarily because women were not aware that the symptoms required treatment. The places a substantial number of untreated symptomatic women at risk for other reproductive issues, like pelvic inflammatory disease, spontaneous abortion, preterm delivery, and human immunodeficiency virus infection [1–5]. Women were more likely to seek care for symptoms if they were married, particularly if they were established in their marriage. Second, we found that women were less likely to report alternative use of informal health providers for RTI specific care, mainly due to a lower rate of pharmacy utilization.

Our observation that most rural Indian women do not seek care for reproductive symptoms due to a lack of knowledge about the severity of RTI symptoms has been reported in past similar research [12]. Since this study was a secondary analysis, we were limited in data available to analyze why some women perceived that RTI symptoms were a legitimate reason to seek treatment, while others did not. This, in combination with the finding that formal education level had no association with health care seeking behavior in our study may reflect prior observations of a reproductive health education deficit in Indian school system [22]. Encouraging research shows that a large majority of Indian adolescents view sexual health education programs to be important, but unfortunately a much smaller percentage receive opportunities to learn about sexual health in school [23]. Further evidence suggests educational interventions led by community health workers that target women of reproductive age and are designed to increase awareness and prevention of RTI symptoms have some potential for changing female reproductive health care related behaviors in India [24–26]. When aiming to increase care seeking behavior for reproductive symptoms, though, it seems that community health programs targeting general education alone, without sex-specific reproductive biology education, may be insufficient for improvements [27].

The only determinant of treatment seeking for reproductive symptoms in our bivariate analysis was marital status. Past literature has established that status as a young, unmarried woman decreases the probability of care seeking behavior for RTI symptoms, and our study confirmed the need for interventions targeting that demographic [14]. We hoped to investigate differences in treatment seeking between newly married women and women who have been established in their marriage, because newly married women tend to be more vulnerable and less autonomous in their own health care decision making process than women who are in established marriages [28, 29]. However, we were limited by small sample size in those groups. General marital status was a significant predictor of increased care seeking behavior, which could reflect greater cultural barriers that unmarried, generally younger women need to overcome in to engage in reproductive health care seeking behavior [14, 30]. Other previously observed patterns in reproductive symptom reporting showed that cultural factors impacting a woman’s autonomy, including accepting any justification for wife beating, lack of contact with natal kin, and having experienced physical abuse are all negatively associated with RTI symptom reporting behavior [31].

General inclination to seek health care for a variety of reasons might also influence RTI care seeking behavior, although we were limited in our ability to examine this due to potential redundancy in the recall of recent health experiences for women experiencing acute RTI symptoms alongside other recent health problems. Yet, access to health care services in rural India is important for achieving health utilization for both general and reproductive symptoms. Notably distance and access to affordable transport to health centers was cited as a determinant of RTI care seeking decisions by 12% of women, with an equal number reporting social barriers from work or domestic duties influencing those decisions. Thus, RTI health knowledge and social conditions tied to sex and gender are at least as important determinants of RTI treatment as health care service accessibility in rural India.

Interestingly, those who sought medical care during their last experience with a health issue were more likely to self-report RTI symptoms when compared to those who chose not to seek medical care. It is possible that women with recent RTI symptoms have a heightened awareness of their health care visits or willingness to use health care for other purposes, but it also seems reasonable to believe that treatment seeking behavior for women with RTI symptoms is over-estimated because the presence of RTI symptoms may be under-reported, particularly in women lacking autonomy. Women have reported feelings of shame and fear of social stigma and repercussions from reporting vaginal symptoms in general, including menstruation. This may have resulted in smaller samples sizes of RTI symptomatic women for some data collection in this study, especially among adolescent girls in fear of being perceived as sexually active. There is potential for future investigation regarding symptom-reporting disparities, especially whether those disparities apply exclusively to RTI symptom reporting or if there are disparities in other symptom reporting as well. Treatment seeking for RTIs is further over-estimated due to the high prevalence of asymptomatic RTIs [16].

Women in our survey used formal providers at a higher rate for RTI symptoms than most recent symptoms of any kind, which reflects that there is not a significant cultural gap in trust of formal providers for treatment of reproductive symptoms. The fact that formal providers were utilized more frequently across all treatment seeking groups highlights positive potential outcomes for interventions aiming to improve RTI symptom treatment by formal providers.

## Conclusions

Multi-pronged interventions are needed to effectively increase health care seeking behavior in response to RTI symptoms in rural Indian women. Perhaps the most essential area where improvement can be made is increasing availability of care providers who are qualified to treat reproductive tract symptoms, an approach that has been enacted in India through mobile clinics. A promising aspect of recent mobile clinic initiatives [32] is that they also include an educational outreach component aimed at increasing women’s awareness of the severity of reproductive tract infection symptoms. Further evaluation is needed on the effectiveness of the mobile clinic approach, and another element that should be added in the future is intentional screening of asymptomatic women. Reproductive treatment outreach programs have been shown to be more effective when recruitment of women is strategically organized by community health workers [33]. Many asymptomatic RTIs that otherwise could go undetected for years can be diagnosed quickly, cheaply, and on-site of a mobile clinic [34]. Unmarried girls and women should be prioritized for both education and treatment programs, as they may be particularly unaware of the significance of symptoms due to lack of sexual activity or less likely to use them. Many people are unaware that RTI symptoms can reflect unsafe hygienic, rather than sexual behaviors. Unmarried women may be less willing to seek gynecological care out of fear that their families or communities will perceive care seeking to be for a sexually transmitted disease, which could harm their prospects of marriage. Unless women are extremely confident in their family’s trust and support, social norms may incentivize women to hide symptoms. Finally, further research should examine the association between autonomy and symptom reporting, along with culturally sensitive interventions designed to increase health care autonomy in women, as other interventions targeted towards increased autonomy have shown promise [35, 36]. RTI treatment seeking is critical for addressing disease burden linked to other adverse reproductive issues, and well-designed interventions have the potential to improve current unacceptable rates amongst rural Indian women.

## Supporting information

S1 Dataset Survey data used in this analysis. (XLS)

S1 Codebook Variable names and definitions for use of the S1 Dataset. (XLSX)

## Abbreviations

UTIs: urinary tract infections
BV: bacterial vaginosis
RTI: reproductive tract infection
STIs: sexually transmitted infections
DHS: District Level Household Surveys
OR: odds ratio
CI: 95% Confidence Interval
